# Respiratory allergies and house dust mites: evaluating public awareness in Riyadh, Saudi Arabia – a cross-sectional study

**DOI:** 10.3389/fmed.2025.1646684

**Published:** 2025-08-18

**Authors:** Samia T. Al-Shouli, Zeyad Kurdee, Sultan Ayoub Meo, Samiah Alhabardi, Abdulaziz Alnasser, Bader Almogbel, Khalid Abdulaziz, Naif Alquwayi, Saad Alhanaya, Sultan Alshehri, Yazeed Hussein, Mohammed Hussein

**Affiliations:** ^1^Immunology Unit, Department of Pathology, College of Medicine, King Saud University, Riyadh, Saudi Arabia; ^2^Clinical Biochemistry Unit, Department of Pathology, College of Medicine, King Saud University, Riyadh, Saudi Arabia; ^3^Department of Physiology, College of Medicine, King Saud University, Riyadh, Saudi Arabia; ^4^Department of Pharmaceutics, College of Pharmacy, King Saud University, Riyadh, Saudi Arabia; ^5^College of Medicine, King Saud University, Riyadh, Saudi Arabia

**Keywords:** house dust mites, awareness, knowledge, education, hygiene, Saudi Arabia

## Abstract

**Objectives:**

Respiratory allergies are a highly challenging and growing public health problem. The house dust mites (HDM) are common allergens that trigger respiratory allergic reactions. This study aimed to investigate the knowledge and attitudes of the general population in Riyadh, Saudi Arabia, regarding HDM-induced respiratory allergy.

**Methods:**

This cross-sectional study recruited a total of 917 subjects, 481 males (52.5%) and 436 females (47.5%). An online questionnaire was developed and underwent a pilot process to assess its clarity, relevance, and comprehensiveness. The questionnaire had 19 questions divided into three parts (sociodemographic, knowledge, and attitudes). The questionnaire was distributed via social media platforms, including email and WhatsApp.

**Results:**

The most common reported allergies were sinusitis (17.1%), asthma (12.4%), rhinitis (11.6%), eczema (6.2%), urticaria (2.5%), rash (1.5%), and conjunctivitis (1.3%). Male participants (mean = 5.11, SD = 2.14) had a significantly lower mean knowledge score than females (mean = 6.03, SD = 1.76), *p* = 0.001. The participants aged 46–59 (mean = 6.05, SD = 1.81) had the highest knowledge (*p* = 0.001), participants with a postgraduate degree (mean = 6.04, SD = 1.86) had the highest knowledge, followed by those with a bachelor’s degree or diploma, high school graduates, and those with less than a high school education (*p* = 0.006). A sizable portion, 47.7% (437) of respondents, correlated HDM-induced respiratory allergies with living environments characterized by poor hygiene and crowded residential places.

**Conclusion:**

The most commonly reported allergies were sinusitis, asthma, rhinitis, eczema, urticaria, rash, and conjunctivitis. The majority of participants had limited knowledge of HDM-induced allergy; male participants demonstrated significantly lower knowledge compared to females. Participants aged 46–59 years who held a postgraduate degree showed the highest level of knowledge. A sizable portion of the participants correlated HDM-induced respiratory allergies with living environments characterized by poor hygiene and crowded residential places.

## Introduction

The increased prevalence of allergies has become a concern over the last couple of decades. It has become a public health issue, ranking among the top three conditions that necessitate a significant effort towards prevention in the 21st century (1). Individuals susceptible to respiratory allergies are often sensitized to multiple allergens (2), whose inhalation triggers airway hyperresponsiveness, inflammation, and tissue damage in the lung airways. The house dust mites (HDM) are the most common allergens that trigger respiratory allergic reactions (3). Allergen nomenclature database records 81 entries for Dermatophagoides farinae, Dermatophagoides microceras, Dermatophagoides pteronyssinus, and Euroglyphus maynei HDM allergens (4).

The older homes, without resident children, single-family homes, and homes with enhanced bedroom humidity, are associated with greater HDM allergen concentration predictors (5). HDM affects approximately 65–130 million people globally, and failure to treat HDM can lead to asthma or atopic dermatitis (6). A combination of genetic susceptibility and exposure to inhaled substances is attributed to respiratory allergies (7). HDM inhalation promotes the activation of the NLRP3 inflammasome in the lungs, which induces caspase-1 and IL-1β in alveolar macrophage maturation (3). HDM exhibit geographical variation in different subspecies, depending on the presence of specific conditions, such as a temperature range of 18–24°C and humidity levels of 70% or higher, to thrive. HDM has been reported from 63 different countries worldwide (8) with a higher prevalence in South and North America, Europe, the Middle East, and the coastal areas of Western Africa and Eastern Asia. HDM, date palm, acacia, and mesquite can cause perennial and seasonal allergies in the Middle East (9). The children (5.6%) and adolescents (14.0%) suffer from rhinoconjunctivitis in Saudi Arabia (10).

HDM two species, Dermatophagoides pteronyssinus and Dermatophagoides farinae, have been reported from Riyadh, Jeddah, Abha, and Qassim regions of Saudi Arabia. These species occur in Jeddah and Abha regions due to favourable climatic conditions. Consequently, residents of Jeddah exhibit the highest sensitivity, while residents of Qassim display the lowest in Saudi Arabia (11). Similarly, Dermatophagoides pteronyssinus, D. farinae, and Blomia tropicalis allergens have been reported more frequently in coastal cities of Saudi Arabia than in non-coastal towns (12). The prevalence of Der p1, Der f1, and Blo t5 allergens was 25, 25, and 17%, respectively, in the Saudi homes. Literature has reported HDM as the most frequent indoor allergen (77.8%) in Saudi Arabia (11–13). Limited literature is available on the awareness of HDM-induced respiratory allergies. This study aimed to assess the knowledge and attitudes on HDM-induced respiratory allergy among the general population in Riyadh.

## Materials and methods

### Study design and settings

This questionnaire-based cross-sectional study was conducted in the Department of Pathology (Immunology Unit), College of Medicine, King Saud University, Riyadh, Saudi Arabia, from May 2024 to December 2024.

### Inclusion and exclusion criteria

The study participants were adults aged 18 years or older, residing in Riyadh, Saudi Arabia, who could read and understand the English-language questionnaire and or Arabic language. They had provided informed consent before participating in the study. The participants who submitted incomplete responses to the questionnaire were excluded from the study.

### HDM questionnaire

Riyadh, the capital of Saudi Arabia, is an urban area with a population of approximately 8 million and an area of 1,798 km^2^, characterized by an arid desert climate. It experiences extreme summer temperatures and mild winter temperatures, with rainfall below 100 mm, and frequent dust storms (14). For this cross-sectional study, we developed an English language and an Arabic language version of an online questionnaire to investigate the knowledge and attitudes of individuals with HDM-induced respiratory allergy. The survey questionnaire consisted of 19 items, including an introductory section for informed consent, whether to contribute to the study, and provided an option for respondents to select their responses. No reward was presented to the contributors, and information was kept confidential.

The non-probability convenience sampling technique was used. The data were collected through an online questionnaire; a Google survey sheet was distributed on social media platforms, including WhatsApp and Instagram, via a link targeted at the population. After a week, we sent a reminder to submit the reply at their convenience. The questionnaire underwent a pilot process among five participants to assess its clarity, relevance, and comprehensiveness. Additionally, two specialists in the field provided feedback on its content and structure. The questionnaire had 19 items, divided into three parts (sociodemographic, knowledge, and attitudes). Participants’ knowledge was evaluated by asking nine questions about various aspects of the topic, and a total score out of nine was calculated. Regarding the assessment of attitudes, a set of statements was administered to participants, and they were asked to indicate whether they agreed or disagreed with each statement. The participants were mainly targeted by community, university groups and interest-based pages related to health, allergies, or general well-being. To enhance representativeness, we distributed the questionnaire survey across groups representing targeted age ranges, educational backgrounds, socioeconomic backgrounds, and geographic locations. Moreover, we posed in both English and Arabic to maximize participation. In this study, a total of 917 participants completed the questionnaire, comprising 481 males and 436 females. The power formula was used to calculate the sample size. The required sample size was 385; however, our sample size in this study exceeded the minimum required sample size needed to detect the effect size at 80% power.

### Statistical analysis

Google Sheets were used to obtain the participants’ responses. The responses file was downloaded to a Microsoft Excel Sheet and analyzed using SPSS IBM version 21.0. The student’s *t*-test was applied to compare two independent groups with continuous dependent variables. For comparisons involving three or more groups, one-way ANOVA was used. The *Post hoc* Tukey test was used to examine differences in mean knowledge scores between high school graduates and those with a postgraduate degree. The significance level in this study was set to 0.05; any *p* < 0.05 was denoted as statistically significant.

## Results

The study subjects were 18 years old. ≤ old residing in Riyadh. A total of 917 participants completed the questionnaire, with 47.5% (436) being female and 52.5% (481) being male. The majority of participants fell within the 18–29 age range, comprising 44.8% of the total sample, followed by the 30–45 age group (28.2%), the 46–59 age group (21.9%), and those aged 60 or older (5.1%). All participants were from Riyadh. Regarding nationality, 87.5% of participants (803) were Saudi, while 12.5% (114) were non-Saudi. When considering educational level, the most significant proportion of participants reported having a diploma or bachelor’s degree (61.1%), followed by high school graduates (23.5%), postgraduate degree holders (11.2%), and individuals with less than a high school education (4%). As for allergic conditions, the most commonly reported allergies were sinusitis, Asthma, Rhinitis, Eczema, Urticaria, Rash and Conjunctivitis (17.1, 12.4, 11.6, 6.2, 2.5, 1.5 and 1.3%), while a majority of participants (62.9%) have no allergies (see Table 1).

**Table 1:** Sociodemographic characteristics of the study participants.

Variables		Frequency	Percentage
Gender	Female	436	47.5%
	Male	481	52.5%
Age	18–29	411	44.8%
	30–45	259	28.2%
	46–59	201	21.9%
	60 or older	46	5.0%
City	Riyadh	917	100%
Nationality	Non-Saudi	114	12.4%
	Saudi	803	87.6%
Education	Less than high school	37	4.0%
	High school graduate	216	23.6%
	Diploma or Bachelor’s degree	561	61.2%
	Postgraduate degree	103	11.2%
Allergies	Rhinitis	106	11.6%
	Sinusitis	157	17.1%
	Eczema	57	6.2%
	Asthma	114	12.4%
	Urticaria	23	2.5%
	Conjunctivitis	12	1.3%
	Rash	14	1.5%
	No allergy	576	62.9%

The lowest possible allergy score was 0 and the highest was 9 with a median of 6. The allergy score distribution peaked at 6. Those who scored above the median accounted for 35.3% of the total participants (see Table 2).

**Table 2:** Distribution of knowledge score, frequency, percentage, and cumulative percentage.

Score	Frequency	Percent	Cumulative percent
0.00	27	2.9	2.9
1.00	17	1.9	4.8
2.00	27	2.9	7.7
3.00	60	6.5	14.3
4.00	118	12.9	27.2
5.00	153	16.7	43.8
6.0	191	20.8	64.7
7.0	171	18.6	83.3
8.0	121	13.2	96.5
9.0	32	3.5	100
Total	917	100.0	

Male participants (Mean = 5.11, SD = 2.14) had a significantly lower mean knowledge score than female participants (Mean = 6.03, SD = 1.76), with a *p*-value of less than 0.001, as determined by a student *t*-test. Knowledge scores varied across different age groups. Participants aged 46–59 (Mean = 6.05, SD = 1.81) had the highest mean knowledge score, followed by those aged 60 or older (Mean = 5.71, SD = 1.99), 30–45 (Mean = 5.80, SD = 2.01), and 18–29 (Mean = 5.12, SD = 2.04). Differences in mean knowledge scores across age groups were statistically significant, with a *p*-value of less than 0.001, as determined by One-Way ANOVA. Non-Saudi Participants (M = 6.14, SD = 1.92) had a significantly higher mean knowledge score than Saudi participants (M = 5.46, SD = 2.02), with a *p*-value of 0.001, as determined by a student *t*-test. This indicates that non-Saudi participants tend to have a higher level of knowledge than Saudi participants.

The mean knowledge scores varied across various levels of education. Participants with a postgraduate degree (M = 6.04, SD = 1.86) had the highest mean knowledge score, followed by those with a diploma or bachelor’s degree (M = 5.60, SD = 2.01), high school graduates (M = 5.22, SD = 2.04), and those with less than a high school education (M = 5.32, SD = 2.16). The differences in mean knowledge scores across education levels were statistically significant, with a *p*-value of 0.006, as determined by a one-way analysis of variance (ANOVA). Additionally, further *post hoc* Tukey tests revealed a statistically significant difference in mean knowledge scores between high school graduates and those with a postgraduate degree. There was no statistically significant difference in mean knowledge scores between participants with allergies (M = 5.68, SD = 1.96) and those without allergies (M = 5.46, SD = 2.05), as determined by a student *t*-test, with a *p*-value of 0.114 (see Table 3).

**Table 3:** Comparison of mean knowledge scores with the socio-demographic characteristics of study subjects.

Variables		Mean	SD	*P*-value
Gender	Female	6.03	1.76	0.001
	Male	5.11	2.14	
Age	18–29	5.12	2.04	0.001
	30–45	5.80	2.01	
	46–59	6.05	1.81	
	60 or older	5.71	1.99	
Nationality	Non-Saudi	6.14	1.92	0.001
	Saudi	5.46	2.02	
Education	Less than high school	5.32	2.16	0.006
	High school graduate	5.22	2.04	
	Diploma or Bachelor’s degree	5.60	2.01	
	Postgraduate degree	6.04	1.86	
Allergy	Rhinitis	5.68	1.96	0.114
	Sinusitis	5.46	2.05	
Overall	Eczema	5.54	2.02	

A significant portion, 47.7% (437) of respondents correlated HDM-induced respiratory allergies with living in environments with poor hygiene. Regarding the relationship between HDM-induced allergy and crowded places, the majority of participants, 49% (449), agreed that people living in densely populated residential areas are more susceptible to HDM-induced allergies. Furthermore, 45.5% (417) of participants believed that HDM-induced allergies were more widespread in working-class regions compared to prestigious areas. Lastly, 45.1% (414) of respondents agreed that field workers are more exposed to HDM-induced allergies than office workers (Table 4).

**Table 4:** Participants attitude towards HDM induced allergy.

Statement	Agree *N* (%)	Disagree *N* (%)	Do not know *N* (%)
Individuals diagnosed with HDM-induced respiratory allergy live in an environment with poor hygiene	437 (47.7%)	294 (32.1%)	186 (20.3%)
People who live in crowded residential places are more exposed to house dust mite-induced allergy	449 (49%)	186 (20.3%)	282 (30.8%)
House dust mite-induced allergy is less widespread in working-class regions compared to prestigious areas	417 (45.5%)	242 (26.4%)	258 (28.1%)
Field workers are more exposed to house dust mite-induced allergy than office workers	414 (45.1%)	233 (25.4%)	270 (29.4%)

## Discussion

This study from Saudi Arabia investigated HDM-induced allergy knowledge and awareness. The subjects comprised 52.5% males and 47.5% females, with the majority of the age group falling within the 18–29 years range. Additionally, 44.8% had attained a diploma or bachelor’s degree. In this study, the most commonly reported allergies were sinusitis, asthma, rhinitis, eczema, urticaria, rash, and conjunctivitis. The majority of participants had limited knowledge of HDM-induced allergy; male participants demonstrated significantly lower knowledge compared to females. Participants aged 46–59 years who held a postgraduate degree showed the highest level of knowledge. A sizable portion of the participants correlated HDM-induced respiratory allergies with living environments characterized by poor hygiene and crowded residential places. The present study findings suggest that the majority of the general population in Riyadh lacks sufficient understanding of HDM-induced respiratory allergies. These results align with other studies indicating low levels of respiratory allergy and asthma knowledge among parents of asthmatic children (15). A prior survey of childhood asthma awareness in Saudi Arabia found that over 50% were aware of the disease, while about 40% had misconceptions about it. Another study on asthma awareness in Saudi Arabia revealed that the Saudi population has limited knowledge about asthma (16, 17).

Al-Harbi et al. (18) reported that respiratory allergy asthma knowledge was higher with increasing age and education. Similarly, the present study also revealed a positive correlation between age and level of education in asthma knowledge. We observed a significant association between participants’ educational attainment and their expertise in managing respiratory allergies caused by house dust mites. Higher educational attainment appears to correlate with greater awareness of this allergen-induced respiratory condition, as evidenced by the high mean percentages among postgraduate degree holders, followed by those with diplomas or bachelor’s degrees, and then by high school participants. Interestingly, it was shown that counselling significantly increased knowledge and awareness of environmental control measures related to dust allergies in Indonesia (19).

Regarding attitudes towards individuals affected by house dust mite-induced respiratory allergies, 49% of participants reported a correlation between living in areas with poor hygiene or crowding and a higher likelihood of developing such allergies (Table 4). Previously, it was established that household dust contains HDM that correlates with eczema and asthma (20). A study regarding house dust mites (HDMs) and their relationship to allergies revealed that 54% of study respondents had good knowledge about HDMs and their association with allergies (21). Our study findings, which align with the existing literature, suggest that a lack of knowledge and a misunderstanding exist regarding HDM allergy. While allergies, in general, have increased in urban and industrialized regions compared to rural areas over the last decades (22), correlating house dust mite-induced respiratory allergies with poor hygiene is inaccurate. We found that some participants believed allergies are more prevalent in working-class areas and among field operatives than in high-class areas and among office workers. Comparable results have indicated that HDM allergies correlate with living environmental standards (23).

### Study strengths and limitations

Awareness of HDM-induced allergies is highly crucial, as these invisible allergens are a leading cause of respiratory health problems, which are often undetected in everyday environments. This study from Saudi Arabia investigates the HDM-induced allergy awareness. The findings are essential for the population, health officials and policymakers. The present study findings are based on data from arid areas, Riyadh, Saudi Arabia, and introduce an essential approach to HDM. This indoor environmental health issue has often been overlooked. This study is necessary for the public, health officials and policymakers as it highlights the hidden public health issue about HDM allergies. HDM respiratory allergies are often undiagnosed and mixed with other causal factors. The findings support a shift from treatment to prevention, highlighting simple and effective home-level interventions such as home hygiene, clean mattresses, carpets, furniture, bed sheets, humidity control, and appropriate and regular cleaning routines. The limitations of this study include the potential for recall bias by using the questionnaire. The cross-sectional nature cannot establish causality between HDM exposure and respiratory allergic outcomes.

## Conclusion

The commonly reported allergies were sinusitis, asthma, rhinitis, eczema, urticaria, rash, and conjunctivitis. The majority of participants had limited knowledge of HDM-induced allergy; female participants demonstrated better knowledge than males. The participants aged 46–59 years who held a postgraduate degree showed the highest level of knowledge. A sizable portion of the participants correlated HDM-induced respiratory allergies with living environments characterized by poor hygiene and crowded residential places. Following the publication of this study, future campaigns in schools, colleges, and universities will be launched, and the study’s findings will be presented at the regional health sciences conference. Moreover, findings will be shared with print media to enhance public knowledge about HDM-induced respiratory allergies.

## Data Availability

Data may be provided on reasonable request to the corresponding author.
